# A New Approach to Homeostatic Regulation: Towards a Unified View of Physiological and Ecological Concepts

**DOI:** 10.1371/journal.pone.0107737

**Published:** 2014-09-23

**Authors:** Cédric L. Meunier, Arne M. Malzahn, Maarten Boersma

**Affiliations:** 1 Alfred-Wegener-Institut, Helmholtz-Zentrum für Polar- und Meeresforschung, Biologische Anstalt Helgoland, Helgoland, Germany; 2 Department of Ecology and Environmental Sciences, Umeå University, Umeå, Sweden; 3 Department of Marine Science and Fisheries, College of Agricultural and Marine Sciences, Sultan Qaboos University, Al-Khod, Sultanate of Oman; The University of Wollongong, Australia

## Abstract

Stoichiometric homeostasis is the ability of an organism to keep its body chemical composition constant, despite varying inputs. Stoichiometric homeostasis therefore constrains the metabolic needs of consumers which in turn often feed on resources not matching these requirements. In a broader context, homeostasis also relates to the capacity of an organism to maintain other biological parameters (e.g. body temperature) at a constant level over ambient environmental variations. Unfortunately, there are discrepancies in the literature and ecological and physiological definitions of homeostasis are disparate and partly contradictory. Here, we address this matter by reviewing the existing knowledge considering two distinct groups, regulators and conformers and, based on examples of thermo- and osmoregulation, we propose a new approach to stoichiometric homeostasis, unifying ecological and physiological concepts. We suggest a simple and precise graphical way to identify regulators and conformers: for any given biological parameter (e.g. nutrient stoichiometry, temperature), a sigmoidal relation between internal and external conditions can be observed for conformers while an inverse sigmoidal response is characteristic of regulators. This new definition and method, based on well-studied physiological mechanisms, unifies ecological and physiological approaches and is a useful tool for understanding how organisms are affected by and affect their environment.

## Intoduction

The concept of stoichiometry was first developed in the field of chemistry and corresponds to the “*art of chemical measurements, which has to deal with the laws according to which substances unite to form chemical compounds*” [1]. Chemical stoichiometry involves a complex of limitations controlling the interactions between chemical elements. Also in biology, there are boundaries to how chemical elements are assembled, especially as they have to function as a living cell. These constraints are described and integrated in the framework of biological stoichiometry, which is very well developed in ecology (ecological stoichiometry, [2]). Taking a mechanistic stoichiometric approach has proven especially useful in the study of the interactions between different trophic levels. The elemental composition of many autotrophs varies substantially among ecosystems in response to different availabilities of key resources such as CO_2_, solar energy and mineral nutrients in the environment [2]. This variability in plants' biochemical composition affects their quality as food for herbivores which, as a result, often have to consume prey not matching their requirements. This mismatch is caused by herbivores' stoichiometric homeostasis i.e. the ability of an organism to keep its body chemical composition constant, and thus ascertaining a stable environment for cellular processes [3]. Stoichiometric homeostatic regulation reflects underlying physiological and biochemical allocations as organisms respond to their surrounding environments [4], and thus the degree of homeostasis may be highly relevant to fitness and to a species' ecological strategy on the one hand [5] and to recycling processes of superfluous material on the other one [6].

Interestingly, the approach of stoichiometric homeostasis developed by Sterner and Elser [2] is very different from the rest of the huge body of literature existing on physiological homeostasis (e.g. temperature or osmotic pressure, [6]–[7]). In a broader context, homeostasis also relates to the capacity of an organism to maintain other biological parameters (e.g. body temperature, pH, osmolality) at a constant level over ambient environmental variations. Moreover, as stated by Sterner and Elser [2] “*Without homeostasis, ecological stoichiometry would be a dull subject*”, stoichiometric homeostasis must therefore be defined very precisely. Where consumers fall on the continuum from non homeostatic to strictly homeostatic has important consequences for consumer–resource interactions, the supply of carbon and nutrients to higher trophic levels, and nutrient recycling [7]–[11]. However, it has been estimated that about one third of organisms characterized as strictly homeostatic using Sterner and Elser's method [2] are misclassified [12]. The main difference between Sterner and Elser's [2] approach and the one used in physiology (e.g. [13]–[14]) lies in the graphical interpretation of organisms' response to environmental conditions: physiological stoichiometric approaches focus on the shape of the curve of e.g. body temperature versus ambient temperature, whereas the ecological approach typically only considers the slope of the response of consumer nutrient stoichiometry to food nutrient stoichiometry, essentially focusing on the middle part of the response curves, while ignoring the edges. On the other hand, we are not aware of any mathematical approaches to describe the response curves of organisms in traditional physiological approaches, whereas this has clearly been done in ecological stoichiometry [2]. Hence, ecological stoichiometry also needs a physiological underpinning to characterize homeostasis, at the same time the physiological approaches need a proper mathematical description of organisms' response to environmental conditions. Here, we address this matter by reviewing the existing knowledge considering primarily the two ends of the spectrum: regulators (homeostatic) and conformers (non-homeostatic). We propose a new approach to stoichiometric homeostasis unifying ecological and physiological concepts and allowing the characterization of regulators and conformers. While conformers are generally considered completely plastic (e.g. autotrophs regarding their nutrient stoichiometry), we suggest and discuss possible boundaries between which their body stoichiometry can fluctuate. We also pose the question whether stoichiometric homeostasis is as beneficial as generally implied [2], and argue that the ability to store nutrients, which results in flexible body stoichiometry, could be the more advantageous strategy under certain conditions. But first, let us consider the existing ways to define the homeostatic ability of an organism.

## Methods

**Figure 1:**
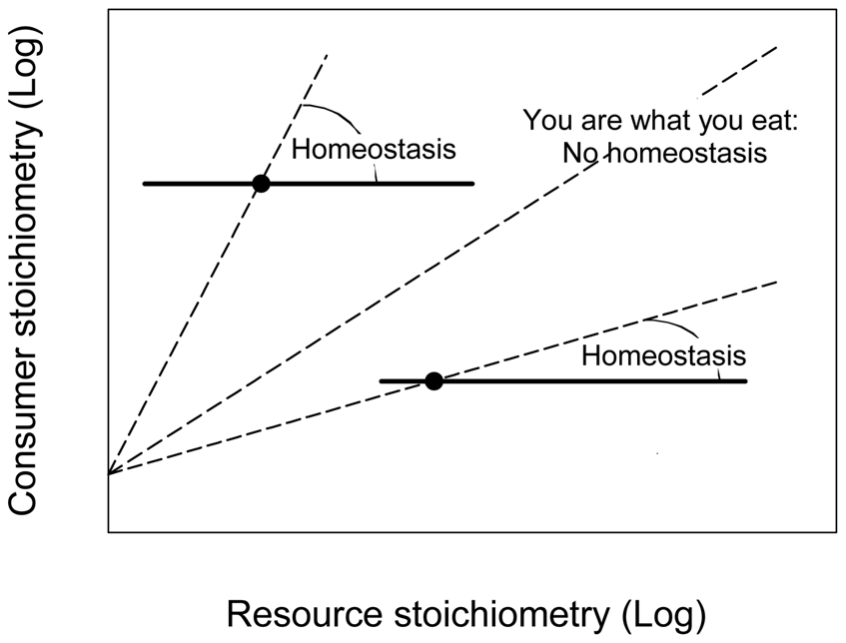
Generalized stoichiometric patterns relating consumer stoichiometry to resource stoichiometry ([2], modified). Horizontal and vertical axes are any single stoichiometric measure, such as N:P ratio. Dashed lines represent no homeostasis, i.e. a constant change of consumer's stoichiometry proportional to the resource stoichiometry. Continuous lines represent strict homeostasis. Sterner and Elser [2] defined that for an arbitrary point x, y in a plot of consumer versus resource stoichiometry, homeostatic regulation of nutrient content x, y can be diagnosed as a slope less than x/y, down to an expected lower limit of zero.

Sterner and Elser [2] proposed graphical as well as mathematical ways to analyze different levels of elemental homeostatic regulation. On a logarithmic plot of consumer versus resource nutrient stoichiometry (mostly ratios of some nutrient to either dry weight or carbon), homeostatic regulation can be diagnosed when the slope is lower than the slope from a constant proportional response (Fig 1): Log y = 1/H * Log x+c, where 1/Η is the slope of the logarithmic plot and Η (eta) the homeostasis coefficient. Η is a regulation coefficient greater or equal than 1. An H value of exactly 1 indicates a complete lack of homeostasis, whereas, as H approaches infinity, the slope of consumer versus resource stoichiometry approaches zero indicating strict homeostasis (Fig. 1). Although this tool has not been extensively used, primarily because it requires a considerable amount of data not readily available, it does allow the identification of important patterns enhancing our understanding of a species' or taxon's role in population dynamics, food webs, and nutrient cycles [12]. For instance, aquatic macro-invertebrates seem to have a more confined nutrient stoichiometry than terrestrial ones and heterotrophs are significantly more homeostatic than autotrophs [12]. The homeostasis definition of Sterner and Elser [2] yields a single number to characterize the degree of homeostasis over the entire range of resource nutrient stoichiometry. It therefore has the severe drawback that it forces the whole range of potential values of x and y in one formula. As such, it does not allow for qualitative changes in the reaction of organism to different ranges of resource nutrient stoichiometry as are implicit in many of the other approaches (e.g. [15]). Since this log-transformation masks small, but ecologically important deviations from a linear relationship, this parameter also simplifies the intrinsic physiology and biochemistry of homeostasis and this may mislead our interpretation of a species role in population dynamics, food webs, and nutrient cycles [12].

**Figure 2:**
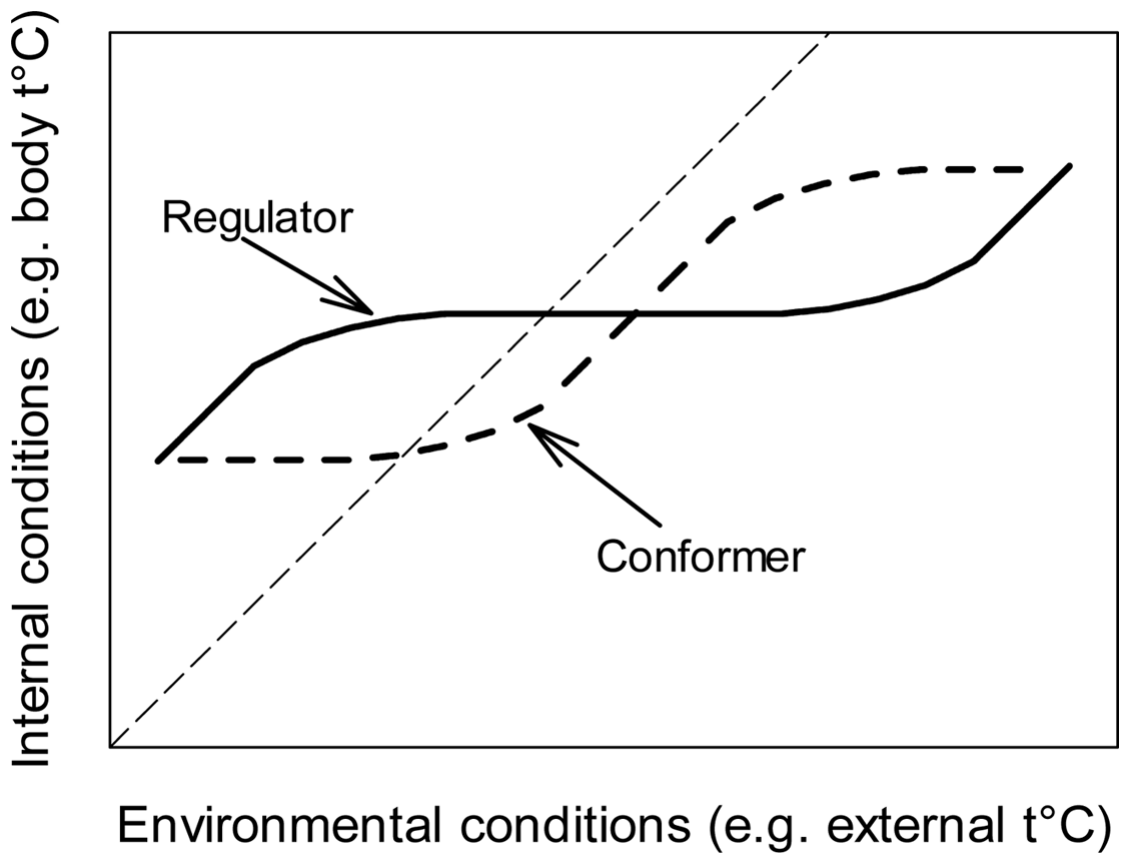
Generalized homeostasis patterns relating organisms internal conditions to external conditions. Regulators maintain their internal composition stable for a certain range of environmental conditions. The homeostasis strength of an organism is defined by the magnitude of the range of environmental conditions over which the internal milieu of the consumer remains stable. Conformers allow the environment to determine their internal composition for a certain range of environmental conditions.

The study of homeostatic regulations is an important research topic in physiology. Regulators keep their internal composition stable for a certain range of environmental conditions while conformers allow the environment to determine their internal composition, up to certain borders. The large amount of data collected, e.g. on thermo- and osmoregulation, allowed physiologists to draw generalizations and highlight that regulators typically exhibit an inverse-sigmoidal response to environmental fluctuations (Fig. 2). In the following, we highlight that a contrasting pattern can be observed for conformers which exhibit a sigmoidal response, and that organisms also present these two types of response in nutrient stoichiometry. In mathematical terms, organisms can be classified when the middle slope of the sigmoid is steeper (conformers) or shallower (regulators) than the two other slopes, anterior and posterior, forming the sigmoid curve (Fig. 2). We identified this pattern using a three segments piecewise regression. The piecewise regression was applied to the datasets using the segmental linear regression method of the software Prism 6 (GraphPad). Our regression model (piecewise) was compared with Sterner and Elser's model (single linear regression) using the Akaike Information Criterion (AIC). Fitting a piecewise regression gives a more accurate representation of the underlying physiological processes than the linear approach of Sterner and Elser [2] as it indicates breaking points below and above which regulators cannot maintain homeostasis anymore. It is important to note that datasets do not always allow the use of three segments because one of them can be out of scope of the experiment. Our method does not use sigmoid curves fit since it would not allow the estimation of the homeostatic strength (see below). We also discuss potential physiological and ecological explanations and implications of these two responses.

## Results and Discussion

### Conformers

**Figure 3:**
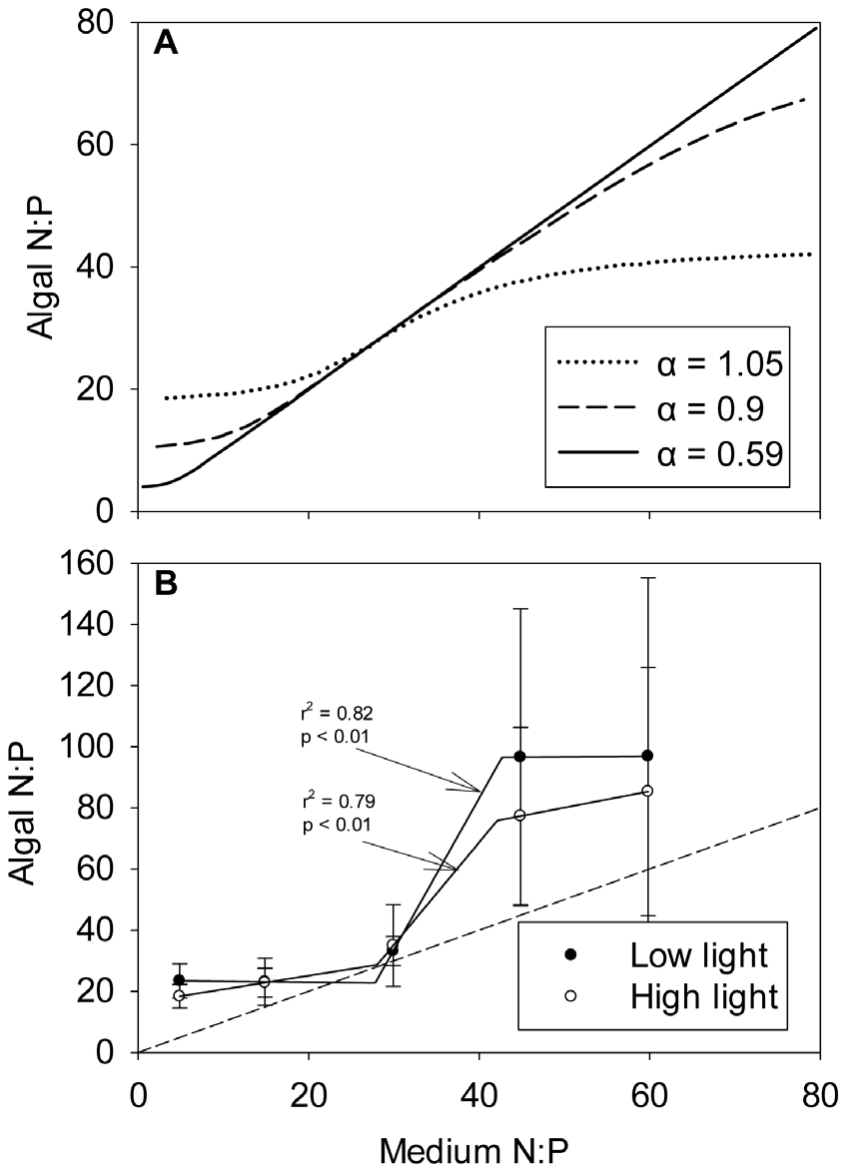
Equilibrium phytoplankton stoichiometry as a function of nutrient supply stoichiometry in chemostats. (A) Klausmeier et al. [15] model predicted phytoplankton N: P under three dilution (growth) rates. The solid line represents Rhee's [36] experimental setup, with dilution (growth) rate α = 0.59 d^−1^. The dashed and dotted lines represent higher dilution rates, α = 0.9 and α = 1.05 d^−1^, respectively. (B) Dependencies of the molar ratios of N:P of *Rhinomonas reticulata* cells at a range of inflow N:P ratios under high light (open symbols) and low light (filled symbols). The dashed-line represent the 1∶1 line and error bars represent standard deviation [17]. Since this example is a clear case but there are only very few data points, the solid line is fitted by eye to the data.

Although elemental composition of autotrophs varies substantially with nutrient availability, it is important to note that their stoichiometry is not solely influenced by nutrient supply ratios [2]. Klausmeier et al. [15] developed a model to explain patterns of phytoplankton composition in chemostat experiments. Their model highlights the importance of growth rate in determining stoichiometry of autotrophs: phytoplankton stoichiometry matches the nutrient supply ratio over a large range for low growth rates, while for higher growth rates, the relation becomes more sigmoidal and phytoplankton stoichiometry varies less (Fig. 3A), indicating a limit to the flexibility of phytoplankton stoichiometry. Thus, phytoplankton stoichiometry matches the nutrient supply stoichiometry over a broad range at low growth rates and over a narrow range at high growth rates, hence illustrating that there are multiple ways to grow slowly but only one to grow fast [16]. Leonardos and Geider [17] made similar observations culturing the cryptophyte *Rhinomonas reticulata* over a range of N:P ratios in chemostats. Although *R. reticulata*'s growth rate was set to a lower value than the one parameterized in Klausmeier et al.'s [15] model, the alga's nutrient ratio did increase non-linearly with increasing nutrient supply ratios (Fig. 3B). Moreover, in a study of seston stoichiometry in Michigan ponds, Hall et al. [18] found a limited flexibility in the N:P ratios of diverse phytoplankton assemblages under widely varying N:P loading ratios. These studies highlight that, due to nutrient storage, phytoplankton stoichiometry is flexible for intermediate nutrient supply ratio but more stable for high and low ones. This case illustrates a particular situation in which the middle slope of the sigmoid is steeper than 1. The organism is accumulating nutrients at a higher rate than it increases in its resource which can happen when the organism is taking up an element at higher rates than it is excreting it. Physiological limits to storage therefore attenuate the elemental flexibility of microalgae [16]. Whereas Klausmeier et al.'s model [15] predicts highly flexible N:P ratios for most of phytoplankton growth rates (ca. 80%), a recent meta-analysis identified strong variance only at the lower 30% of observed growth rates [16]. It is thus likely that the ability to store excess nutrients is restricted to stagnant and slow growth, whereas there is only a limited range of elemental composition allowing fast growth.

**Figure 4:**
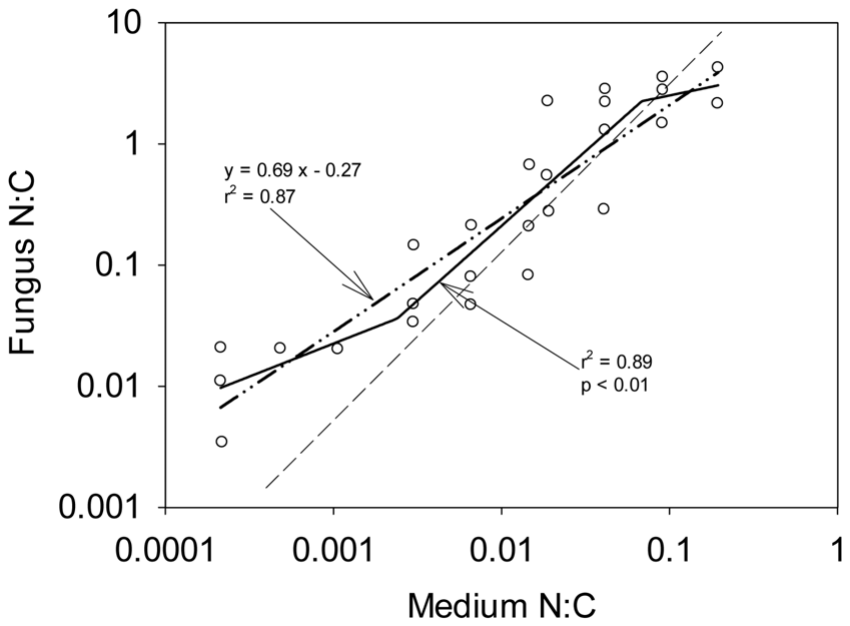
N:C of the fungus *Polyporus versicolor* as a function of medium N:C, data from Levi and Cowling [19]. The dashed line represents the 1∶1 line. The dashed-dotted line represents the linear regression (the equation is indicated in the graph) used by Sterner and Elser [2]. The solid line represents the three segments piecewise regression applied to the data. R-square and p-value are shown on the plot. Although the ΔAIC (−2.27) indicated that a straight linear regression fits better the data than a piecewise regression, the residuals are not distributed homogenously around the line. We therefore plotted the piecewise regression.

Even though Klausmeier et al.'s model [15] was first developed for phytoplankton, one may expect the same response in other conformers since the underlying physiological mechanisms should be similar. A wood-consuming fungus raised in synthetic medium over a range of N:C ratios (Fig. 4, [19]) was classified as regulator by Sterner and Elser [2] since the slope of the linear regression was less than 1. However, when fitting the model of Klausmeier et al. (Fig. 4, [15]) to these data, we observe that the fungus is a conformer rather than a regulator and that the results can be interpreted much in the same way as we did above for phytoplankton. Even though the difference between the single linear and the piecewise regressions AIC (ΔAIC = −2.27) indicates that the first model is the more likely one, the linear regression cannot approximate the data points well because the residuals are not distributed homogenously around the line. This emphasizes the importance of a precise and accurate definition of homeostasis in order to properly describe organism's response to environmental fluctuations.

**Figure 5:**
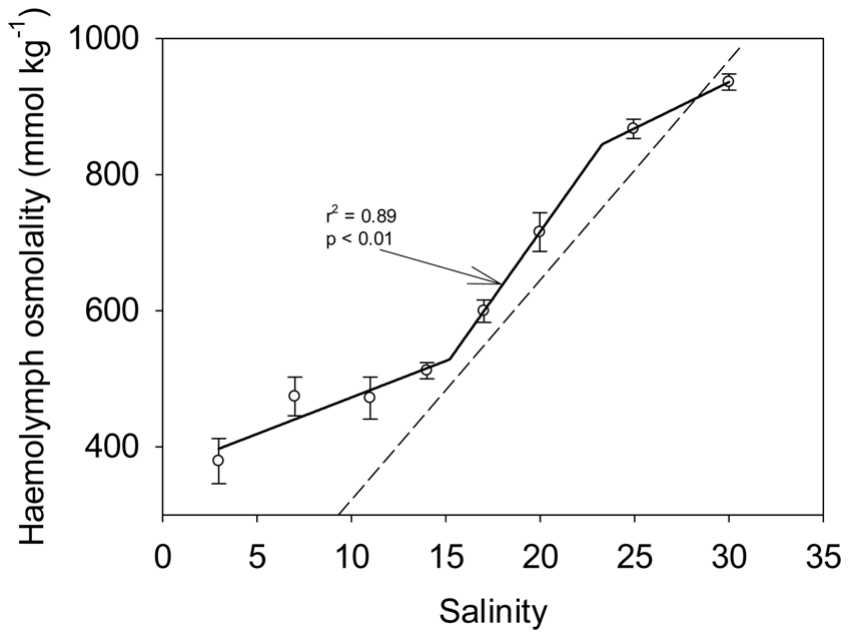
Haemolymph osmolality of the isopod *Idotea chelipes* in relation to the medium osmolality. Data represent means and error bars represent standard deviation of five observations [20]. The dashed line represents the 1∶1 isosmotic line. The solid line represents the three segments piecewise regression applied to the data. R-square and p-value are shown on the plot. Although the ΔAIC (−10.20) indicated that a straight linear regression fits better the data than a piecewise regression, the residuals are not distributed homogenously around the line. We therefore plotted the piecewise regression.

**Figure 6:**
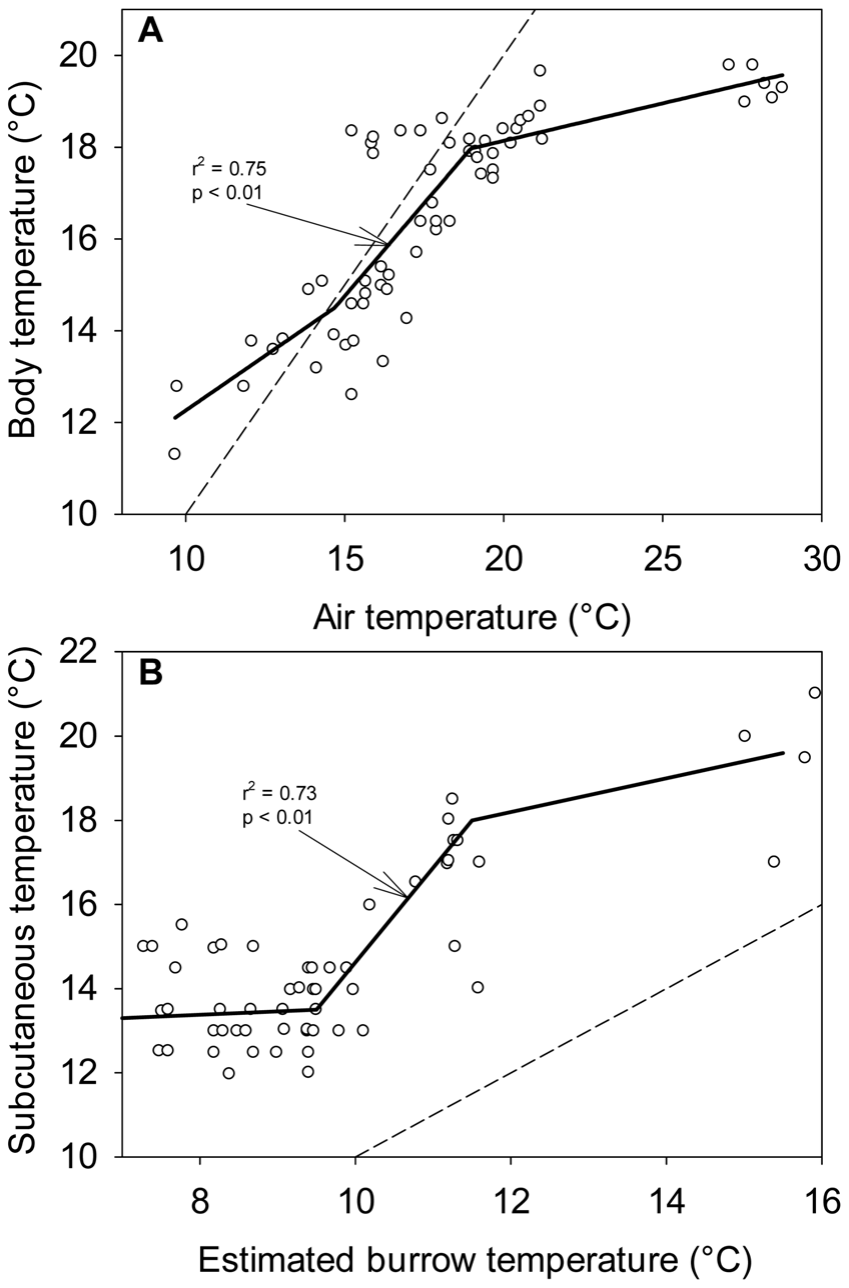
Relationship between organismal temperature and external temperature. (A) Relationship of body temperature of the toad *Odontophrynus occidentalis* and substrate temperature in the dry season [21]. (B) Relationship between torpid subcutaneous temperature in the pichi *Zaedyus pichiy* and burrow temperature, estimated as the average temperature measured 1 m aboveground for the previous 10 days [23]. Dashed lines represent 1∶1 lines and solid lines represent threesegments piecewise regressions applied to the data. R-square and p-values are shown on the plot. The ΔAIC (16.68 and 16.58) indicated that piecewise regressions fit better the data than a straight line.

The pattern of sigmoidal response can also be observed for a large variety of conformers with respect to different traits under regulation. Łapucki and Normant [20] acclimated an isopod to a range of salinities and measured the effect of this treatment on the organism's osmolality (Fig. 5). Although the organisms responded in the same way as the fungus of Levi and Cowling [19], the authors, unlike Elser and Sterner [2], characterized them as conformers highlighting a huge discrepancy in data interpretation between ecologists and physiologists. As for the previous case (Fig. 4), the ΔAIC (−10.20) indicates that the best model is a linear regression but again the residuals are also not distributed homogenously around the line. We therefore recommend to apply a piecewise regression to such datasets. Similarly, Sanabria et al. [21] measured fluctuations of body temperature of a toad as a function of external temperature and observed a sigmoidal response of this conformer (Fig. 6A). Interestingly, even regulators can occasionally behave as conformers. For instance, hibernation and daily torpor are physiological strategies to cope with energetic challenges characterized by a decreased physiological activity resulting in reduced body temperature and rate of metabolism [22]. Superina and Boily [23] studied variations in body temperature of a dwarf armadillo or pichi (*Zaedyus pichiy*) during hibernation and recorded the same sigmoidal response (Fig. 6B), characteristic of a non-homeostatic response to environmental fluctuations. Hence, as shown by the sigmoidal pattern, in order to reduce metabolic costs, conformers do not regulate their internal milieu for intermediate environmental conditions, which can be considered the comfort zone, but actively regulate at high and low e.g. temperature to survive. The same mechanism could explain the sigmoidal response of phytoplankton stoichiometric changes with changes in nutrient supply. Thus, we hypothesize that conformers do not regulate their body composition to save energy and store nutrients but, as a survival mechanism, they keep their internal milieu constant at extremer high and low environmental conditions. This can be linked to the intensity and duration of environmental changes relative to the organism's growth rate. Montechiaro and Giordano [24] hypothesized that natural selection should favour the best compromise between minimizing the cost of adjusting to environmental changes and growth rates maximization. The authors further stated that this compromise also depends on the time required to obtain a reproductive advantage from acclimation, thus on growth rate. Hence, for organisms growing fast and living in a patchy environment, prone to strong fluctuations, the most advantageous strategy is to be conformer (see [25]).

### Regulators

While thermo- and osmoregulators keep their internal milieu constant in order to function effectively in a broad range of environmental conditions [22], the benefits of stoichiometric homeostasis are less obvious [12]. While for example, polar zooplankton experience high food abundance during the short spring/summer period and convert it into depot lipids (resulting in fluctuating stoichiometry, [25]), tropical and subtropical plankton have a year round low-level supply of food available, and thus accumulate lipid reserves to a much lower degree [26]. Båmstedt [27] postulated that differences in lipid accumulation reflect different reproductive strategies, as the nutritional energy acquired is invested to produce offspring. In the tropics, organisms have faster growth rates and short reproductive cycles without accumulation of significant reserves. In polar regions, on the other hand, slower growth rates and long reproductive cycles predominate. This suggests a coupling between storage strategy and the degree of homeostasis, implying that regulators may not actively maintain their body nutrient composition but instead do not store nutrients. Moreover, the absence of storage also implies that these nutrients can be used only at fixed, stoichiometric proportions. As a result, a decrease in the supply of nutrient X will lead to a decrease in the use of nutrient Y without affecting the stoichiometric X:Y body composition [28].

We suggest a new graphical method, inspired by the way physiologists define homeostasis, to characterize regulators. While the ecological stoichiometric view concentrates only on the slope of consumer versus resource stoichiometry [2], physiologists consider two distinct parameters. The first one is the “homeostatic strength” and can be characterized as the range of environmental conditions over which an organism maintains its internal environment constant (Fig. 2, e.g. [13]–[14]). The second one is the “homeostatic capacity” and can be defined as the slope of consumer versus resource stoichiometry in the ‘flat’ section of the curve. As in the approach of Sterner and Elser [2], the closer the slope is to 0, the stronger the homeostatic capacity is. Since we hypothesized that organisms exhibiting a stable stoichiometry are not able to store nutrients, the first parameter corresponds to the range of resource nutrient ratio over which the organism can function properly while the second one can be interpreted as the nutrient storage capacity. Moreover, Sterner and Elser's model does not consider the breaking points at which an organism cannot function properly anymore and the subsequent (sub) lethal points. Their model assumes one homeostasis across the complete resource gradient. The available evidence suggests that it might be more accurate to assess homeostasis separately within regions of the resource gradient.

**Figure 7:**
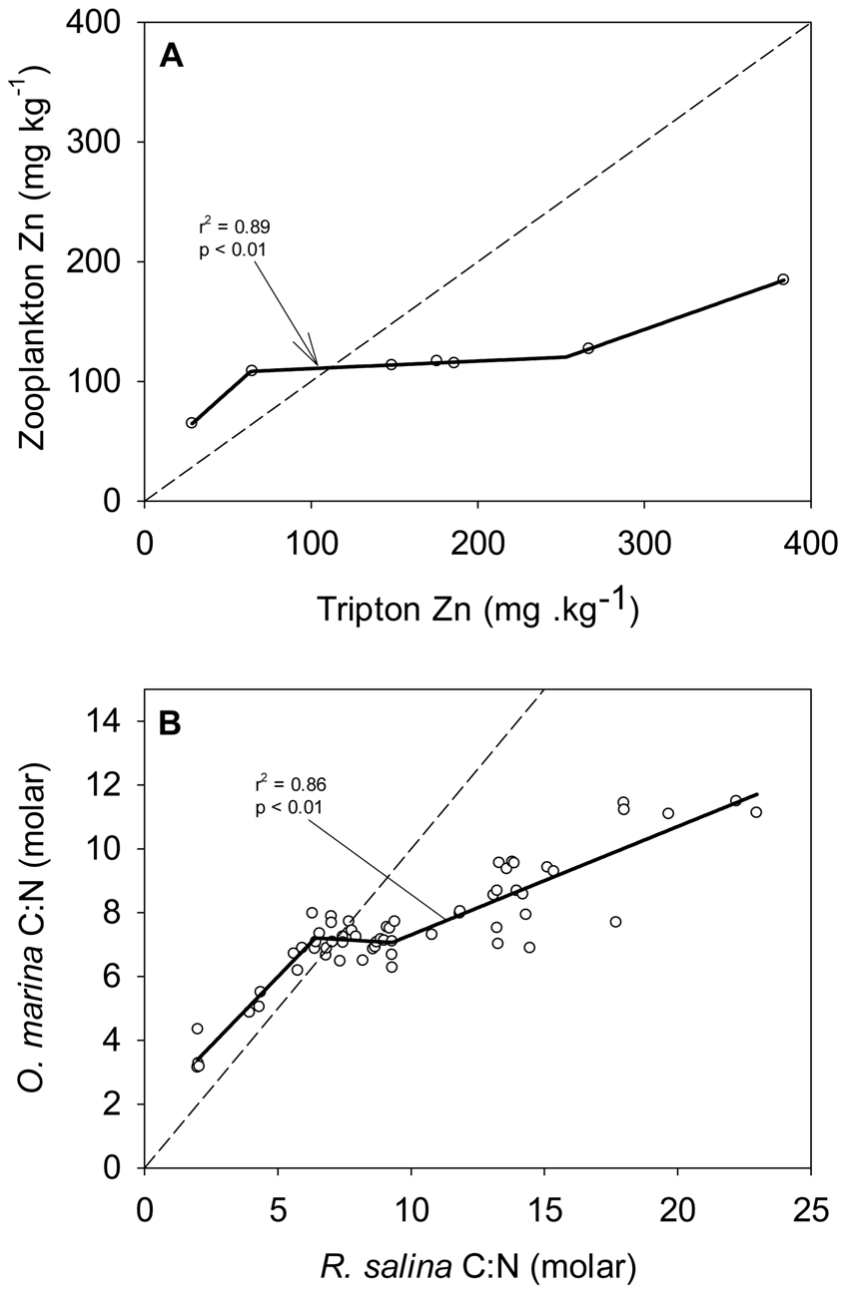
Relationship between consumer and resource nutrient content. (A) Zinc content in zooplankton and tripton in lakes [29]. The dashed line represents the 1∶1 line and the black line is fitted by eye to the data due to the very low number of data points. (B) C:N of the dinoflagellate *Oxyrrhis marina* as a function of its prey *Rhodomonas salina* C:N (unpublished data from Meunier et al.). The dashed line represents the 1∶1 line. Solid lines represent the three segments piecewise regression applied to the data. R-square and p-value are shown on the plot. The ΔAIC (23.97) indicated that a piecewise regression fits better the data than a straight line.

The large amount of data collected on thermo- and osmoregulators allowed physiologists to draw generalizations and highlight that regulators typically exhibit an inverse-sigmoidal response to environmental fluctuations (Fig. 2, [22]). Unfortunately, in ecological stoichiometry there is a paucity of appropriate datasets on organisms' nutrient stoichiometry, which limits our ability to make broad conclusions. Further studies should include larger stoichiometric gradients, and measure more points across that gradient to accurately characterize non-linearity and break points. Sterner and Elser (2002) analyzed the dataset of Zauke at al. [29], who measured the concentrations of zinc in freshwater zooplankton and their food, and characterized these organisms as close to strictly homeostatic (Fig. 7A). While the zooplankton zinc content was stable in the intermediate range of resource stoichiometry, at very low and very high zinc content in the resource, homeostatic regulation breaks down. Even though zinc is an essential requirement for a healthy body, excess zinc can be harmful, and cause zinc toxicity thus justifying the need to regulate tightly its concentration [30]. Further, we tested the effect of a large gradient of resource stoichiometry on herbivore stoichiometry. We fed the herbivorous dinoflagellate *Oxyrrhis marina* with the alga *Rhodomonas salina* of various C:N (methods and analytic procedure described in [31]) and indeed observed an inverse-sigmoidal response (Fig. 7B). The plateau for *O. marina* is relatively short indicating that this dinoflagellate has a weak homeostasis strength which can be explained by its feeding behaviour (see below “Mild homeostasis”, [31]). These examples suggest that the reason why the inverse-sigmoidal response characteristic of regulators is rarely observed in the context of stoichiometry (in contrast to temperature and osmoregulation) is because it occurs only at extremely low and high resource stoichiometry. However, this response is ecologically relevant and reflects breaking points below and above which homeostasis cannot be maintained anymore.

### Mild homeostasis

Not surprisingly, interspecific and intertaxa differences in the degree of homeostasis can be observed. According to the definition of homeostasis we previously described, an organism can have a mild homeostatic (1) strength or (2) capacity.

(1) Unlike planktonic metazoans, which possess a high homeostatic strength [32]–[33], protozoans have a mild homeostasis strength which results from the ingestion and assimilation of food in excess. This behaviour, which is typically adopted after a period of starvation (see below, [31]), leads to the accumulation of certain nutrients in the body hence changing the organism's stoichiometry. For instance, Droop [34] recorded that protists replenished their nutrient stores after a period of nutrient limitation by raising the uptake of the previously limiting nutrient once it becomes available again, but also that feeding went beyond this simple replenishment. This strategy, which results in considerable body stoichiometry flexibility, allows the organisms to function efficiently when its diet meets its needs relatively well and to store a particular element, when available in excess, in order to be prepared for future nutrient limitation [31], [35]. Flexible nutrient content provides protozoans a greater capability to persist under nutrient limitation than occurs among other regulators [7]. Animals also integrate resources across high- and low-quality diets, their long-term growth can therefore be greater than predicted from diet-specific growth rates. The type of integration could be predicted from the degree of stoichiometric homeostasis. Species with mild homeostatic strength exhibit a capability for resource integration providing an advantage in heterogeneous environments [25]. Moreover, there might be a trade-off to the degree of homeostasis strength between the life span of an organism and the heterogeneity of the environment in which it lives. Long lived organisms could indeed afford occasional growth depressions, while few hours in an imbalanced tide pool means a lot for a protozoan such as *Oxyrrhis marina*.

(2) Following our definition of homeostasis, an organism can also have a mild homeostatic capacity. This second parameter corresponds to the definition of the lack of homeostasis developed by Sterner and Elser [2] i.e. when the slope of consumer versus resource stoichiometry approaches one (Fig. 1). However, even if the middle plateau is not strictly horizontal, an organism should be classified as regulator if the shape of the response to environmental fluctuations follows the inverse-sigmoidal type. This way of characterizing organisms has been adopted two decades ago by physiologists. For example, clear patterns of hemolymph osmoregulation in aquatic crustacean have been defined [13]. As a matter of fact, estuarine and shore crabs exhibit limited capabilities of regulation and the osmolality of the hemolymph decreases with the isosmotic line. Whether that pattern has to be considered as reflecting a failure of the regulation system or as a strategy to reduce the diffusion gradients is a question of interpretation. Interestingly, limited capabilities for regulation will influence the natural distribution of the weak regulator species. For instance, species which show such limited capabilities are typically found or even live for prolonged periods in fluctuating environments like estuaries and tide pools [13]. Hence, there is a strong link between the degree of homeostasis of an organism and the heterogeneity of the environment in which it lives.

## Conclusions and Outlook

In this paper, we highlighted patterns in organisms' response to environmental fluctuations and offered new definitions of stoichiometric homeostasis. While autotrophs were until now considered non-homeostatic we highlight that there are boundaries between which their body stoichiometry can fluctuate [16]. These boundaries are tighter at higher growth rates which suggests that physiological limits to storage attenuate the elemental flexibility of microalgae. On a plot of internal body versus external conditions, a slope of 0 indicates that the nutrient stoichiometry of an organism remains unchanged while a slope higher than 0 indicate an accumulation of nutrient. A slope higher than 1 indicates that the organism is accumulating nutrients at a higher rate than it increases in its resource. This can happen when the organism is consuming an element at higher rates than it is excreting it. On such a plot, conformers typically exhibit a sigmoidal response to external fluctuations. We hypothesize that conformers do not regulate their body composition for intermediate external conditions to save energy or store nutrients and that, as survival mechanism, they keep their internal milieu constant at high and low environmental conditions. Graphical tools can also be used to identify regulators, which display an inverse-sigmoidal response to environmental variations. We hypothesize that the stable stoichiometry of those organisms is due to, unlike e.g. microalgae, the absence of nutrient storage. Further, when investigating how an organism' stoichiometry is affected by its resource, we show the need to consider a range of resource stoichiometry as large as possible in order to identify the breaking points which are necessary to categorize properly different degrees of homeostasis. This response is ecologically relevant and highlights breaking points below and above which homeostasis cannot be maintained anymore. This new method based on well-studied physiological mechanisms is the first to unify ecological and physiological approaches, which were until now disparate and partly contradictory. This is a useful tool allowing a better understanding of how organisms are affected by and affect their environment. While stoichiometric homeostasis was until now considered beneficial [2], it appears from our study that to store nutrients, which results in flexible body stoichiometry, is a more advantageous strategy.
